# Sequential targeting biomimetic nano platform for enhanced mild photothermal therapy and chemotherapy of tumor

**DOI:** 10.1016/j.csbj.2023.04.024

**Published:** 2023-04-26

**Authors:** Lianfu Wang, Manxiang Wu, Yuning Pan, Dong Xie, Chengyuan Hong, Jianbin Li, Xuehua Ma, Huachun Xu, Huayu Li, Tianxiang Chen, Aiguo Wu, Qiang Li

**Affiliations:** a Department of Radiology, The Affiliated People’s Hospital, Ningbo University, Ningbo 315040, China; b Cixi Institute of Biomedical Engineering, International Cooperation Base of Biomedical Materials Technology and Application, Chinese Academy of Science (CAS) Key Laboratory of Magnetic Materials and Devices, Zhejiang Engineering Research Center for Biomedical Materials, Ningbo Institute of Materials Technology and Engineering, CAS, 1219 Zhongguan West Road, Ningbo 315201, China; c Department of Radiology, The First Affiliated Hospital of Ningbo University, Ningbo 315010, China; d Department of Mechanical, Materials and Manufacturing Engineering, University of Nottingham Ningbo China, Ningbo 315100, China; e Advanced Energy Science and Technology Guangdong Laboratory, Huizhou 516000, China

**Keywords:** Triple negative breast cancer, Cell membranes, Photothermal therapy, Chemotherapy, Polydopamine

## Abstract

Tumor targeting drug delivery is of significant importance for the treatment of triple negative breast cancer (TNBC) considering the presence of appreciable amount of tumor matrix and the absence of effective targets on the tumor cells. Hence in this study, a new therapeutic multifunctional nanoplatform with improved TNBC targeting ability and efficacy was constructed and used for therapy of TNBC. Specifically, curcumin loaded mesoporous polydopamine (mPDA/Cur) nanoparticles were synthesized. Thereafter, manganese dioxide (MnO_2_) and a hybrid of cancer-associated fibroblasts (CAFs) membranes as well as cancer cell membranes were sequentially coated on the surface of mPDA/Cur to obtain mPDA/Cur@M/CM. It was found that two distinct kinds of cell membranes were able to endow the nano platform with homologous targeting ability, thereby achieving accurate delivery of drugs. Nanoparticles gathered in the tumor matrix can loosen the tumor matrix *via* the photothermal effect mediated by mPDA to rupture the physical barrier of tumor, which is conducive to the penetration and targeting of drugs to tumor cells in the deep tissues. Moreover, the existence of curcumin, MnO_2_ and mPDA was able to promote the apoptosis of cancer cells by promoting increased cytotoxicity, enhanced Fenton-like reaction, and thermal damage, respectively. Overall, both *in vitro* and *in vivo* results showed that the designed biomimetic nanoplatform could significantly inhibit the tumor growth and thus provide an efficient novel therapeutic strategy for TNBC.

## Graphical Abstract

**Figure:**
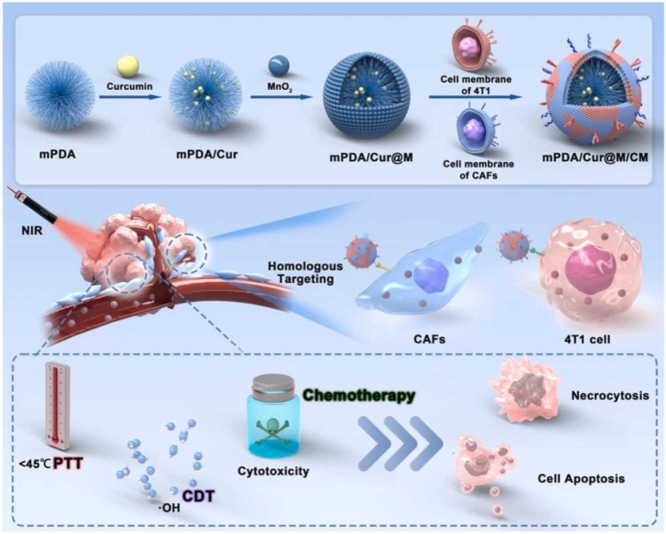

## 1 Introduction

The triple-negative breast cancers (TNBC) is a highly aggressive subtype of breast cancers and has the poorest prognosis in comparison to other subtypes of breast cancer. At present, surgery, radio-therapy, chemotherapy, and immunology therapy are widely used for the management of TNBC, but these strategies are far from satisfactory. Moreover, current targeted treatments for TNBC are limited primarily as a result of high molecular heterogeneity and deficiency of specific targets [1]. Therefore, exploration of effective targeted treatment strategies could markedly improve therapeutic response and improve overall patient survival. Recently, tumor microenvironment (TME) has been widely linked to prognosis and metastasis in TNBC, thus, it has been proposed as a potential target for TNBC therapy [2], [3]. Cancer associated fibroblasts (CAFs) constitute the most abundant population of tumor stromal cells in the TME and can form a barrier which can effectively restricts drugs and immune cells from entering tumor tissue [4], [5]. Therefore, for tumors rich in CAFs, development of novel strategy to target both CAFs and tumor cells remains an urgent problem to be solved to improve anti-tumor efficacy.

Up to now, conventional targeting strategies have been mainly divided into two distinct categories, one is passive targeting strategy *via* enhanced permeation and retention (EPR) effect, whereas the other is active targeting strategy *via* specific ligands, but they all suffer from the disadvantages of low targeting efficiency, complex preparation and high cost [6], [7], [8]. Furthermore, for the multiple surface proteins targeting, several ligands modified on the surface of nanoparticles probably do not lead to the synergistic effect on target receptor binding but can enhance the rate of capture by reticuloendothelial system [9]. In comparison with the conventional targeting strategies, the biomimetic technology of the cell membrane is not only simple in process, but can also lead to improved biocompatibility, good homologous targeting and immune escape through the preservation of the whole membrane protein [10], [11]. Specifically, the biomimetic nanoparticles can exhibit cell-mimicking properties through camouflaging the synthetic cores with a layer of cell membrane derived from the various natural cells, such as erythrocytes [12], cancer cells [13], platelets [14], macrophages [15], stem cells [16], neutrophils [17] and bacterial components [18], which are endowed with dual functions of internal materials and natural cells. However, for some tumors with complex microenvironment such as TNBC and pancreatic cancer, single cell membrane modification might not be able to achieve effective drug delivery [19], [20]. Of note, hybrid cell membranes coated nanoparticles have shown significant advantages in integrating of multiple functions and cascade targeting [21], [22], [23]. Although they have been applied for enhancing the drug accumulation at the target sites and caused significant inhibition of tumor growth, the ability for hybridization of CAFs and cancer cell membrane for the sequential delivery in TNBC therapy has been rarely reported.

In view of the inferior heat resistance of tumor cells in comparison with the normal cells, photothermal therapy (PTT) serves as an effective local anti-tumor treatment modality [24], [25]. In addition, PTT can substantially loosen the tumor matrix and normalize tumor hardness by damaging CAFs [26], [27]. Therefore, CAFs-targeting photothermal strategy is considered as a promising candidate for enhanced cancer treatment. However, high temperature photothermal therapy can cause substantial damage to the normal tissues [28]. It has been established that the down-regulation of heat shock protein (HSP) can significantly reduce the thermal tolerance of cells, and thus mild temperature PTT combined with HSP inhibitor can achieve ideal anti-tumor effect and protect the healthy tissue and cells from thermal damage [29], [30], [31]. Curcumin as a natural polyphenol compound extracted from ginger plants, which has been widely used in various malignancies *via* inhibiting tumor cell proliferation, down-regulating oncogenic transcription factors, and inhibiting tumor angiogenesis [32], [33]. Notably, curcumin can inhibit the function of HSP90 by combining with HSP90 and promoting the dissociation of HSP90 from its co-molecular chaperone P23 [34], [35]. Therefore, the strategy of curcumin used in combination with photothermal therapy could be useful to achieve synergistic antitumor effect at lower temperature. In order to further improve the anti-tumor efficacy, different treatment strategies have been flow combined to obtain therapeutic benefits. Interestingly, regulating the oxidative stress state of tumor tissues through chemodynamic therapy (CDT) can effectively kill tumor cells by damaging lipids, proteins and DNA [36]. On the contrary, treatment regimens which can target both CAFs and tumor cells simultaneously, as well as their subsequent anti-tumor effects have rarely been studied.

**Scheme 1:**
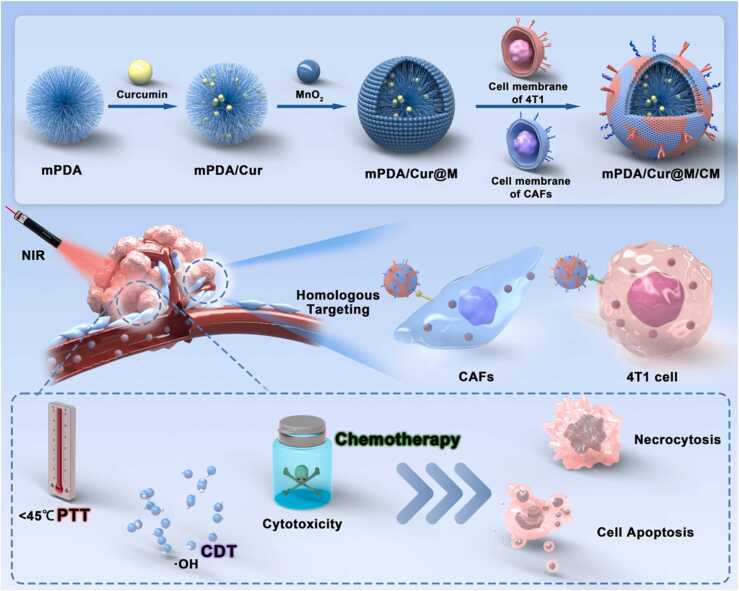
(A) Synthetic scheme of hybrid cell membrane camouflaged core-shell structure nanosystem (mPDA/Cur@M/CM). (B) Illustrated mechanism of mPDA/Cur@M/CM mediated tumor chemotherapy, CDT and PTT.

In this study, we have proposed the hybrid cell membrane camouflaged nano platform to simultaneously exhibit the combined effects of PTT, chemotherapy and CDT for synergistic anti-TNBC therapy. Mesoporous polydopamine (mPDA) was used as the nano core based on its good biosafety, photothermal conversion performance and drug loading capacity. Thus, considering that strategy of down regulating the heat resistance of the tumor cells is conducive to killing the tumor tissues and protecting healthy tissues at low temperatures, curcumin, a component of herbal, was selected as an anti-tumor drug and a heat shock protein inhibitor loaded in mPDA. Thereafter, manganese dioxide shell was formed by chemical method in the outer layer of mesoporous polydopamine, which could be effectively reduced to Mn^2+^ in the environment rich in glutathione, and hence can display substantial antitumor effects through Fenton like reaction. Finally, the mixed membrane of the tumor cells and CAFs were wrapped on the surface of the above materials, so that the nano system was endowed with the ability to specifically target both the tumor cells and CAFs in an orderly fashion through homologous targeting (Scheme 1). To sum up, this study demonstrated an effective anti-cancer strategy to realize sequential targeting for mild PTT, chemotherapy and CDT by utilizing a mixture of CAFs and breast cancer cells membranes camouflaged nano platform, which could provide a novel treatment strategy for clinical management of TNBC in the future.

## 2 Experimental section

### 2.1 Materials

Dopamine hydrochloride, 1,3,5-Trimethylbenzene (TMB) (97 %, A.R.)and pluronic F127 (98 %, A.R.) were bought from Aladdin Chemistry Co. KMnO_4_ (≥99 %, A.R.) were bought from Sinopharm Chemical Reagent Co. Fibroblast activation protein antibody (FAP1) (AF5344, 50 μL) and α-smooth muscle actin antibody (α-SMA) (BF9212, 50 μL) were purchased from Affinity. Recombinant mouse TGF-β1 protein (7666-MB-005/CF, 5 μg) was purchased from RD systems. Ultra-Low adhesion (ULA) U-bottom 96-well plates (174925) were purchased from Thermofisher Scientific Inc. HSP90 antibody (AH732, 40 μL) and membrane protein extraction kit (P0033, 100 T) were bought from Beyotime Biotechnology Co. Calcein-AM/Propidium Iodide Live/Dead Cell Double Staining Kit (CA1630, 500 T) was bought from Beijing Solarbio Science & Technology Co.

### 2.2 Preparation of mPDA

The mPDA nanoparticles were synthesized by following the procedure of the previously reported method with slightly modifications [37]. Briefly, the pluronic F127 (0.45 g), TMB (0.8 mL) and the dopamine hydrochloride (0.3 g) were sequentially added into the mixture of H_2_O (10 mL) and ethanol (10 mL) followed by constant stirring for 30 min. Then, the pH value of the above mixture was adjusted to 8 with ammonia to initiate polymerization and subsequently stirring for 3 h at the room temperature. After the mixture was stirred for 3 h at room temperature, mPDA nanoparticles obtained were washed with a mixture of deionized water and ethanol (v/v=1:1) and stored at 4 °C for further use.

### 2.3 Preparation of the cell membrane

The cell membrane was extracted by the previously reported method [38]. The normal fibroblasts were first cultured and after reaching 70 % of confluence, TGF-β1 (100.0 ng/ mL) was added to promote the transformation of the normal fibroblasts into CAFs. Thereafter, to obtain the cell membrane of CAFs, the membrane protein extraction kit was used. Briefly, the collected cells were dispersed in a mixture of 1 mL membrane protein extraction buffer solution A and 1 mM phenylmethanesulfonyl fluoride (PMSF), were then incubated an ice bath for 10–15 min. The above mixture was processed with freezing–thawing method thrice to break up CAFs, followed by centrifugation at 700 g for 10 min. The precipitate was next discarded for removal of the nucleus and unbroken cells. The obtained supernatant was further centrifuged at 14 000 g for 30 min and then the precipitation was collected and stored at −20 °C for further use. The membrane of 4T1 cells were extracted using at the same method.

### 2.4 Preparation of mPDA/Cur@M/CM

First, 12 mg curcumin and 4 mg mPDA were dispersed 5 mL deionized water followed by stirring at the room temperature for 24 h. The mPDA/Cur was collected *via* centrifugation (1000 rpm, 15 min) and washed with the deionized water thrice. Secondly, 30 mg potassium permanganate and 10 mg mPDA/Cur were added into 10 mL deionized water under sonication for 30 min. The centrifugation and washing of the sediment was then conducted thrice to obtain mPDA/Cur@M. Thereafter, to achieve cell membrane coated mPDA/Cur@M, 500 μL mPDA/Cur@M (2 mg/mL), 250 μL cell membrane of CAF (1 mg/mL) and 250 μL 0.5 mg cell membrane of 4T1 (1 mg/mL) were mixed, and the solution was extruded 30 times through 400 nm polycarbonate porous membranes using an Avanti mini-extruder (Avanti Polar Lipids, USA). Finally, the mPDA/Cur@M/CM was collected *via* centrifugation and dispersed in the deionized water for further experiments. The preparation method of mPDA/IR808 @M/CM was the same with mPDA/Cur@M/CM, but IR808 were used instead of curcumin.

### 2.5 SDS−PAGE

The membrane protein concentration of CAFs and 4T1 cells were detected by employing the BCA assay kit (Beyotime, China). The cell membrane of CAFs, 4T1 as well as mPDA/Cur@M/CM were respectively mixed with the loading buffer were heated at 100 °C for 5 min. Afterward, the various samples with equal protein amounts were added into each well of 12.0 % sodium dodecyl sulfate polyacrylamide gel electrophoresis (SDS−PAGE) gels and run at 90 V. The obtained gels were boiled in the deionized water for 3 min, vibrated for 5 min, subsequently stained with Coomassie blue fast dye solution for 10 min and then decolorized with deionized water for 10 min to visualize the different protein bands.

### 2.6 Drug loading capacity

The drug loading rate of curcumin was detected based on a method described in the previous studies with slight modification [39], [40]. Briefly, 4 mg of mPDA/Cur was dispersed in a mixed solution of the deionized water and ethanol (volume ratio 1:1) with a pH value of 4.5. After shaking for 24 h, the supernatant was collected and the concentration of curcumin was detected by UV–visible spectrophotometer.

### 2.7 *In vitro* photothermal performance

*In vitro* photothermal performance of mPDA/Cur@M/CM was evaluated by using digital photothermal imaging system. The photothermal conversion ability of mPDA/Cur@M/CM was investigated by performing the laser irradiation (808 nm, 0.4 W/cm^2^, 10 min) was performed at a set of concentrations (0–500 μg/mL), and the temperature variation was monitored and recorded.

### 2.8 Fenton-like reaction

The hydroxyl radicals (·OH) formed in the reaction were was detected by methylene blue (MB). Briefly, mPDA/Cur@M/CM was mixed with glutathione (GSH) solution (1 mmol/L) and exposed to the laser irradiation for 10 min. Afterwards, the MB solution (containing 25 mmol/L HCO^3−^, 8 mmol/L H_2_O_2_ and 4 μg/mL MB) was added into the above solution. Finally, the mixture was incubated at 37 °C for 30 min, and the absorbance was analyzed by the UV–Vis absorbance intensity at 665 nm to evaluate the rate of MB degradation.

### 2.9 Measurement of intracellular ROS

To further clarify the chemodynamic effect of MnO_2_ shell, DCFH-DA probe was used to visualize the ROS generation. Firstly, CAFs and 4T1 cells were respectively seeded in 6-wells plates and cultured for 24 h. Then, the cells were incubated with mPDA/Cur, mPDA/Cur@M or mPDA/Cur@M/CM for 4 h before DCFH-DA probe (10 μmol/L) was added. The cells were fixed and incubated for 30 min, after which the nucleus were stained with DAPI. Finally, the ROS generation was observed *via* confocal laser scanning microscopy (CLSM).

### 2.10 *In vitro* cellular uptake behaviors

The cellular uptake behaviors of mPDA/Cur@M and mPDA/Cur@M/CM was analyzed by CLSM and flow cytometric analyses. Briefly, CAFs and 4T1 cells (3 × 10^5^ cells per well) were respectively seeded in 6-well plates. When the density of the cells reached 80 %, mPDA/Cur@M or mPDA/Cur@M/CM (50 µg/mL of curcumin) was added into the wells and incubated for 4 h at 37 °C. Thereafter, the medium were removed and the intracellular fluorescence intensity was measured by flow cytometer. Similarly, in CLSM cellular uptake experiment, CAFs and 4T1 cells were respectively seeded in the cell culture dishes. Then, mPDA/Cur@M or mPDA/Cur@M/CM was added into the dishes and incubated for 4 h at 37 °C. Finally, the medium were removed and cellular uptake was visualized by CLSM.

### 2.11 Drug penetration assay

A 3 dimensional (3D) multicellular spheroids (MCS) was constructed and used as an *in vitro* model to investigate the penetrating ability of mPDA/Cur@M/CM. Briefly, the mixture of CAFs and 4T1 cells (7.5 × 10^3^/well and 2.5 × 10^3^/well, respectively) were seeded on ultra-low adhesion U-bottom plates and cultured at 37 °C within a 5 % CO_2_ atmosphere. After 7 days, upon maturation of these 3D spheroids, they were treated with curcumin, mPDA/Cur@M, mPDA/Cur@M/CM, curcumin+laser, mPDA/Cur@M+laser, and mPDA/Cur@M/CM+laser (50 μg/mL curcumin), respectively. These tumor spheroids were washed, fixed, and then observed by CLSM.

### 2.12 Toxicity assay

*In vitro* cytotoxicity of curcumin, mPDA/Cur@M and mPDA/Cur@M/CM with or without laser irradiation were carried out by the CCK-8 assay and staining of live/dead cells. Firstly, CAFs and 4T1 cells were seeded in 96-well plates at a density of 1 × 10^4^ cells per well and cultured at 37 °C for 24 h. Then, these cells were added with mPDA/Cur@M/CM (the equivalent concentration of curcumin ranging from 30 to 70 μg/mL) and received 808 nm laser irradiation (0.4 W/cm^2^, 10 min) after cultured for 4 h. A day later, 10 μL CCK-8 solution was added into each well and incubated for about 2 h. The absorbance of each well at 450 nm was assessed by a microplate reader. Similarly, the curcumin, mPDA/Cur@M or mPDA/Cur@M/CM (50 μg/mL curcumin) was added into 96-well plates with or without laser irradiation, and washed three times with cold PBS after 24 h of co-culture. The cell viability was quantified using an CCK-8 assay.

### 2.13 Live/Dead cell staining assay

The CAFs or 4T1 cells were seeded in the confocal culture dishes at a density of 3 × 10^5^ cells per dish and cultured overnight. Next, curcumin, mPDA/Cur@M or mPDA/Cur@M/CM were added in each dish with or without laser irradiation (808 nm, 0.4 W/cm^2^, 10 min). After 24 h, the cells were washed thrice with PBS and stained with calcein-AM and PI to observe the viable (green fluorescence) and dead (green fluorescence) cells.

### 2.14 Western blotting

NIH-3T3 cells (2 × 10^5^ cells per well) were cultured in a 6-well plate overnight and were then subjected to TGF-β1 (100.0 ng/ mL) treatment or not. After that, these cells were harvested and protein concentration was measured by BCA protein assay kit. The primary antibodies including α-SMA (1:1000; BF9212; Affinity), FAP (1:500; AF5344; Affinity) and β-actin (1:1000; AF2811; Beyotime), as well as secondary antibodies were incubated in accordance to standard protocols. ImageJ software was used to perform semi-quantitative analysis of the protein band.

CAFs and 4T1 cells were implanted in confocal culture dishes at a density of 5 × 10^5^ cells per dish overnight. Thereafter, the cells were treated with curcumin, mPDA/Cur@M, mPDA/Cur@M/CM, curcumin+laser, mPDA/Cur@M+laser, and mPDA/Cur@M/CM+laser (50 μg/mL curcumin), respectively. Thereafter, proteins were extracted from these cells, and then incubated with the primary antibody HSP90 (1:1000, AF5368; Affinity) as well as secondary antibody, respectively. At the end, ECL western blotting detection kit was used to visualize the different proteins.

### 2.15 Animal models

Female Balb/c mice (20–22 g) were purchased from Beijing Vital River Laboratory Animal Technology Co., Ltd. All animal experiments were performed in accordance with instructions of the Chinese Guidelines on Laboratory Animals Use and Care, and the study protocol was approved by Ningbo University Laboratory Animal Center (China) [permit no. (Zhe) 2019–0005]. The mixture of CAFs and 4T1 cells (1 × 10^6^ and 3 × 10^6^ respectively) suspended in 50 μL of PBS were subcutaneously injected into the right side of the back of the mouse to establish a xenograft tumor model. When the volume of the tumor-bearing mouse reached ∼100 mm^3^, the *in vivo* near infrared thermal imaging, fluorescence imaging and cancer therapy experiments were performed under *in vivo* conditions. There were 66 mice used in this study including 3 mice in each group for imaging experiment and 5 mice in each group for anti-tumor evaluation.

### 2.16 *In vivo* targeting and *ex vivo* biodistribution

The *in vivo* targeting performance and biodistribution of the nanoparticles was visualized by both fluorescence imaging and magnetic resonance imaging. For fluorescence imaging experiments, IR808 was used as a model drug. Briefly, tumor-bearing mice were anesthetized using 2 % sevoflurane in oxygen and then injected with mPDA@IR808/M or mPDA@IR808/M/CM through the tail vein. Then, fluorescence imaging was captured and analyzed at the certain time points by an IVIS imaging system. At the end time point, the umor-bearing mice in each group were sacrificed, and thereafter the momentous tissues and organs (tumors, heart, livers, lungs, spleen, and kidneys) were extracted for *ex vivo* fluorescence imaging and semi-quantitative analysis. For the magnetic resonance imaging experiments, the images of tumor-bearing mice were acquired on a 3.0 T clinical magnetic resonance imaging scanner with a small animal imaging coil.

### 2.17 *In vivo* photothermal therapy

PBS, curcumin, mPDA@M, mPDA/Cur@M and mPDA/Cur@M/CM were injected *via* the tail vein, respectively. After 8 h, laser irradiation (808 nm, 0.4 W/cm^2^) was operated at the tumor site for 10 min, and the temperature was monitored every 30 s by using an infrared thermometer.

### 2.18 *In vivo* anti-tumor study

In order to further evaluate the *in vivo* therapeutic efficacy, the tumor bearing mice were randomly divided into nine groups (saline, curcumin, mPDA@M, mPDA/Cur@M, mPDA/Cur@M/CM, curcumin+laser, mPDA@M+laser, mPDA/Cur@M+laser, mPDA/Cur@M/CM+laser) when the mean volume of the tumors approximately reached to 100 mm^3^. The treatment was performed every 2 days. The mice of the laser groups received 808 nm laser irradiation (0.4 W/cm^2^, 10 min) at 8 h after intravenous administration. The volume of the tumors and body weight of mice were monitored every 2 days for 2 weeks. At the end of experiment, all the mice were euthanized. The tumor tissues of all the groups were excised and fixed with 4.0 % paraformaldehyde for H&E staining as per the standard protocol.

### 2.19 *In vivo* safety evaluation

To evaluate the *in vivo* safety of the nanoparticles, mice were euthanized and the level of white blood cell (WBC), red blood cell (RBC), hemoglobin (HGB), platelet (PLT), alanine aminotransferase (ALT), aspartate aminotransferase (AST), blood urea nitrogen (BUN) and creatinine (CRE) of all the groups were detected *via* the blood routine and biochemistry examinations. Then, the major organs of all the groups were removed and hematoxylin and eosin (H&E) staining was conducted for the histological analysis.

### 2.20 Immunofluorescence staining

For immunofluorescence (IF) staining, the tumor tissues of all the groups were harvested and sliced into 5 µm thickness. Then, the IF staining of HSP90, ki67 and TUNEL were performed in accordance with the standard procedures.

### 2.21 Statistical analysis

All the data has been presented as mean± standard deviation (SD) and analyzed by Prism 8.4 (GraphPad Software). The differences were considered statistically as significant at p < 0.05 (*) p < 0.01 (**) and p < 0.001 (***).

## 3 Results and discussion

### 3.1 Preparation and characterization of mPDA/Cur@M/CM

**Fig. 1:**
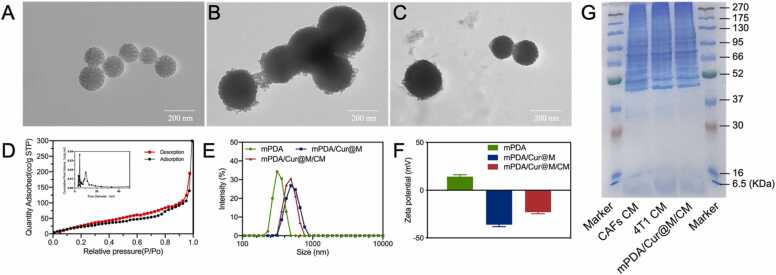
Characterizations of mPDA/Cur@M/CM. The TEM images of mPDA (A), mPDA/Cur@M (B) and mPDA/Cur@M/CM (C). The pore-size distribution curve (inset) and N2 adsorption/desorption isotherms of result of mPDA (D). The size distribution histogram (E) and zeta potential (F) of mPDA/Cur@M/CM. (G) SDS-PAGE protein analysis of marker, CAFs membrane (CAFs CM), 4T1 cells membrane (4T1 CM), and mPDA/Cur@M/CM.

mPDA nanoparticles were synthesized by self-polymerization of dopamine in the presence of TMB and pluronic F127, O_2_ and ammonia. The TEM image, Brunauer–Emmett–Teller (BET), dynamic light scattering (DLS), and zeta potential results of mPDA have been shown in Fig. 1A, D-F, which clearly suggested that mPDA nanoparticles exhibited uniform morphology, regular pore, size distribution (389.6 ± 19.5 nm) and good dispersibility. The Brunauer–Emmett–Teller (BET) surface area and average pore size of mPDA was 101.498 m^2^/g and 18.27 nm respectively. Thereafter, manganese dioxide shell was modified on the surface of mPDA to form mPDA/Cur@M (Fig. 1B), in which the mesoporous structure of mPDA disappeared in the TEM image. Based on X-ray photoelectron spectroscopy (XPS) analysis, the mPDA/Cur@M mainly displayed four distinct peaks of C 1 s, N 1 s, O 1 s and Mn 2p (Fig. S1A). Then the Mn 2p XPS spectrum was separated into divided peaks. The peaks at 650.85 and 638.9 eV are Mn(IV) 2p2/3 and Mn(IV) 2p1/2, respectively (Fig. S1B), thus confirming the surface MnO_2_ formation. In order to acquire orderly targeting capacity, a mixture of the cell membrane of cancer and cancer associated fibroblasts cells was selected to modify the mPDA/Cur@M to form mPDA/Cur@M/CM. It was observed that in comparison with mPDA/Cur@M, smoother and low contrast contour could be observed in the TEM image of mPDA/Cur@M/CM (Fig. 1C), indicating the cell membrane was successfully coated on the surface of mPDA/Cur@M. In addition, compared with mPDA/Cur@M, the hydrodynamic diameter of mPDA/Cur@M/CM was increased from 403.5 ± 4.1–653.5 ± 20.2 nm (Fig. 1E). Moreover, the surface zeta potential of mPDA, mPDA/Cur@M and mPDA/Cur@M/CM was found to be 14.2 ± 2.1, − 36 ± 2.1 and − 22.9 ± 1.7 mV, respectively, which could be attributed to the surface modification of mPDA (Fig. 1F). Next, the expression of marker proteins on the surface of NIH/3T3 cells were detected to identify the successfully construction of CAFs. The results showed the α-SMA expression of NIH/3T3 cells was remarkably increased after stimulation by TGF-α (Fig. S2). Similarly, the FAP1 expression of NIH/3T3 cells was obviously increased after stimulated by TGF-α (Fig. S3). The significantly increase of α-SMA and FAP1 represented the successful construction of CAFs cells. To further verify the existence of the cell membrane on the surface of mPDA/Cur@M/CM, SDS-PAGE was performed. As shown in Fig. 1G, the mPDA/Cur@M/CM retained the characteristic proteins inherited from the cell membranes of both CAFs and 4T1. Additionally, the loading efficiencies of curcumin were raised from 32.6 wt. % to 73.3 wt. % when the feeding ratio of curcumin was raised form 0.5–3, but showed a slightly decrease when the feeding ratio was 4 (Fig. S4).

### 3.2 Fenton-like reaction and *in vitro* photothermal ability

**Fig. 2:**
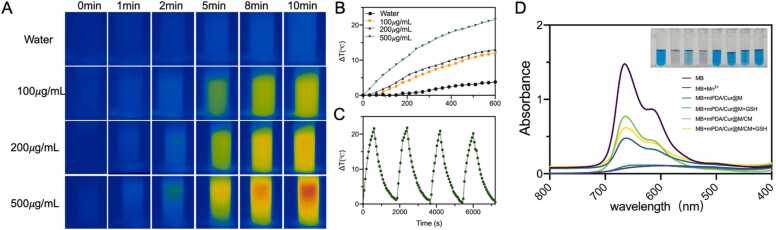
*In vitro* experiments with mPDA/Cur@M/CM. *In vitro* near-infrared thermography image (A), temperature rise curve (B) and photothermal cycle curve of mPDA/Cur@M/CM (C) with laser irradiation (808 nm, 0.4 W/cm^2^, 10 min). Ultraviolet absorption spectra and photographs of MB after incubated with mPDA/Cur@M/CM in the presence or absence of 1 mmol/L GSH for 30 min (D).

Next, considering the good photothermal conversion ability of polydopamine, the *in vitro* photothermal effect of mPDA/Cur@M/CM at the various concentrations were evaluated under 808 nm laser irradiation (0.4 W/cm^2^, 10 min). The *in vitro* near-infrared thermography images of mPDA/Cur@M/CM with the different concentration have been shown in Fig. 2A. The temperature of mPDA/Cur@M/CM NPs (500 μg/mL of mPDA) was increased by 15 °C after irradiation 5 min and finally raised 21.6 °C, but the temperature of the deionized water was just increased to 1.4 °C and 3.8 °C at the same time points (Fig. 2B). Additionally, the results indicated that showed the photothermal effect of mPDA/Cur@M/CM could be positively correlated with the concentration, and even with laser irradiation at low power (0.4 W/cm^2^), the temperature can meet the need of PTT under both *in vitro and in vivo* settings. Thereafter, the laser on/off cycles were performed to evaluate the potential photothermal stability of mPDA/Cur@M/CM. The result showed that photothermal conversion ability of mPDA/Cur@M/CM was not significantly reduced in four laser on/off cycles, thus indicating the mPDA/Cur@M/CM has good photothermal stability (Fig. 2C).

It has been established that manganese dioxide (MnO_2_) can serve as a smart chemodynamic agent for enhanced CDT of cancer through reaction with intracellular GSH to produce Fenton-like Mn^2+^, thereby resulting in the disruption of the cellular antioxidant defense system (ADS) and a large amount of •OH was yielded from endogenous H_2_O_2_ in the presence of physiological HCO^3-^ [41]. To evaluate *in vitro* Fenton-like reaction of mPDA/Cur@M/CM, methylene blue (MB) was selected as an •OH detection probe. It was observed that compared with MB solution, the color and characteristic absorbance of both MB+Mn^2+^ solution and MB+mPDA/Cur@M solution in the presence of GSH disappeared almost completely, indicating the Fenton-like reaction (Fig. 2D). Whereas, mPDA/Cur@M/CM solution in the presence or absence of GSH failed to induce the Fenton-like reaction (all solutions indicated above contained HCO^3-^ and H_2_O_2_). These results indicated that the mPDA/Cur@M with MnO_2_ layer can effectively generate Mn^2+^ in the presence of GSH achieving excellent Fenton-like activity. However, the cell membrane of mPDA/Cur@M/CM may be detrimental to the generation of Mn^2+^ *via* blocking the potential contact between MnO_2_ layer and the external solution, which further suggested that the cell membrane was successfully wrapped on the surface of mPDA/Cur@M. To further clarify the chemodynamic effect of MnO_2_ shell, DCFH-DA probe was used to visualize the ROS generation. The results depicted that the green fluorescence intensity of CAFs and 4T1 cells decreased substantially in the order of mPDA/Cur@M, mPDA/Cur@M/CM, and mPDA/Cur, indicating that the introduction of MnO_2_ shell layer could aid to generate excessive ROS *in vitro*. The level of ROS slightly decreased in both mPDA/Cur@M/CM groups which might partly contribute to membrane modification delay the interaction between MnO_2_ shell layer and the external environment (Fig. S5).

### 3.3 Cellular uptake and penetrating capacity of mPDA/Cur@M/CM

**Fig. 3:**
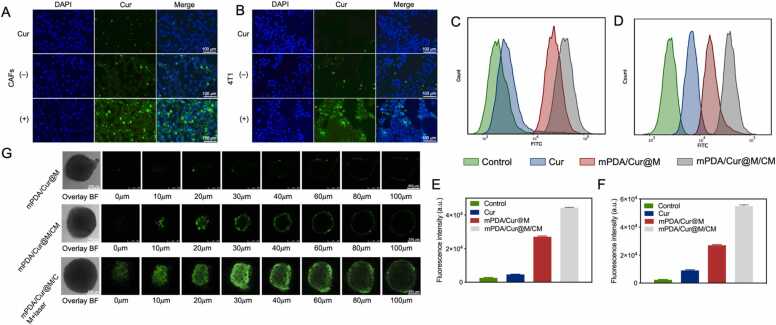
Cellular uptake and penetrating capacity of mPDA/Cur@M/CM. Cellular uptake of curcumin, mPDA/Cur@M and mPDA/Cur@M/CM NPs (containing 50 μg/mL curcumin) in CAFs (A) and 4T1 (B) cell lines, scale bar = 100 µm. Flow cytometry analysis of CAFs (C) and 4T1 (D) cells incubated with different nanoparticles (Cur, mPDA/Cur@M and mPDA/Cur@M/CM) for 4 h and corresponding semi-quantitative analysis of fluorescence intensity processed (E, F) by Image J software (Curcumin concentration: 50 μg/mL). (G) Representative penetrating fluorescent images of MCS at 4 h after treated with mPDA/Cur@M, mPDA/Cur@M/CM and mPDA/Cur@M/CM + laser using CLSM, respectively, scale bar = 250 µm.

A large number of studies have reported that the cell membrane modification can effectively endow the endowed nanomaterials with long internal circulation time and targeting performance through stimulating immune escape and homologous targeting [38], [42]. Thereafter, for verifying the targeting capacity of mPDA/Cur@M/CM, the cellular internalization behavior of mPDA/Cur@M/CM was observed by CLSM and flow cytometric analysis. Upon incubating with CAFs and 4T1 cells for 4 h, respectively, the green fluorescence of both mPDA/Cur@M/CM groups were found to be significantly higher than that of curcumin and mPDA/Cur@M groups (Fig. 3 A, B). As revealed by confocal imaging, the flow cytometry results indicated that the mean fluorescence intensity of curcumin within 4T1 cells in mPDA/Cur@M/CM group was 9.5 and 2.08-fold higher than that in Cur and mPDA/Cur@M groups (Fig. 3 C, E). Meanwhile, the mean fluorescence intensity of Cur within CAF cells was 9.52 and 1.64-fold higher than that in Cur and mPDA/Cur@M as measured by the semi-quantitative analysis (Fig. 3D, F). The above results manifested that the modification of mixed cell membrane can effectively facilitate NPs uptake by CAFs and 4T1 cells, which was consistent with the previous studies.

The penetrating capacity of mPDA/Cur@M/CM was evaluated in the MCS model, which was visualized by CLSM after 4 h incubation (Fig. 3 G). The green fluorescence signals of curcumin in the Cur group was found to be almost invisible. However, compared with mPDA/Cur@M/CM plus laser irradiation, the green fluorescence signals of mPDA/Cur@M/CM began to decline with the Z-axis over 20 µm, but the fluorescence signals from mPDA/Cur@M/CM plus laser irradiation were significantly stronger in comparison to those obtained from free curcumin and mPDA/Cur@M/CM treated groups. These *in vitro* results validated the effective penetrating capability of mPDA/Cur@M/CM in MCS model, which could be mainly credited to the multicell membrane modification and laser irradiation.

### 3.4 Anti-tumor efficiency of mPDA/Cur@M/CM *in vitro*

**Fig. 4:**
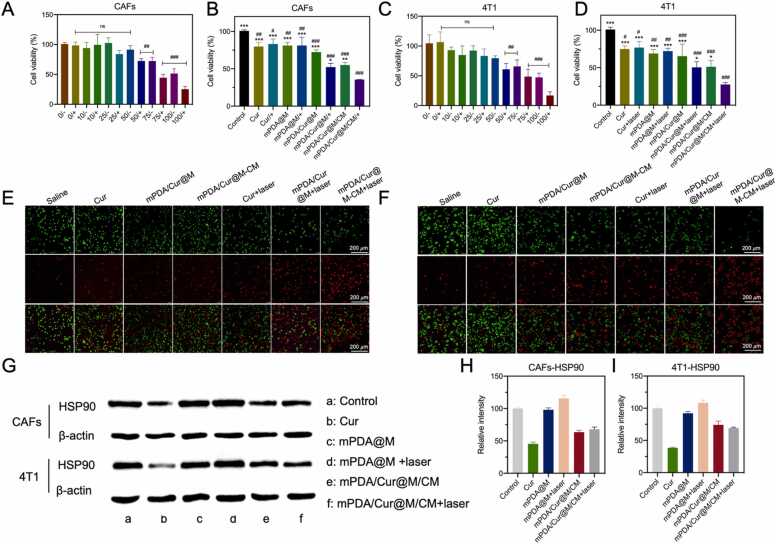
Synergistic *in vitro* cancer inhibition effects of PTT and chemotherapy strategy mediated by mPDA/Cur@M/CM. (A) *In vitro* viability of CAFs treated with mPDA/Cur@M/CM at different curcumin concentrations with or without laser irradiation (808 nm, 0.4 W/cm^2^, 10 min) (A). Viability of CAFs cells received different treatment (B). *In vitro* viability of 4T1 cells treated with mPDA/Cur@M/CM containing different curcumin concentrations in the presence or absence of laser irradiation (808 nm, 0.4 W/cm^2^, 10 min) (C). Viability of 4T1 cells received different treatment (D), n = 4, ∗p < 0.05, ∗∗p < 0.01, ∗∗∗p < 0.001 *versus* the mPDA/Cur@M/CM+laser group. # p < 0.05, ## p < 0.01, ### p < 0.001 *versus* the control group, ns is not significant. Representative fluorescence images of CAFs (E) and 4T1 (F) cells stained with calcein AM (green fluorescence) and propidium iodide (red fluorescence), scale bar = 200 µm. Western blots analysis of HSP90 expression (G) and corresponding quantification in CAFs (H) and 4T1 (I) cells after treated with PBS, Cur, mPDA/Cur@M/CM, Cur+laser and mPDA/Cur@M/CM + laser at the designed doses for 24 h.

To evaluate the anti-tumor efficiency of nanoparticles *in vitro*, mPDA/Cur@M/CM in different concentrations with or without laser irradiation were co-incubated with CAFs or 4T1 cells for 24 h, respectively (Fig. 4 A, C). The results showed that mPDA/Cur@M/CM at low concentration (<50 μg/mL) did not significantly affect the cell viabilities with or without their irradiation by laser (808 nm, 0.4 W/cm^2^, 5 min). At the concentration of 50 μg/mL, the non-laser group did not cause obvious toxicity to CAFs and 4T1 cells, while the laser group significantly inhibited the growth of both kind of cells. When the concentration of mPDA/Cur@M/CM further increased, it exhibited significant cytotoxicity even with or without irradiation. Thereafter, CAFs and 4T1 cells were subjected to different treatments (curcumin is 50 μg/mL, mPDA@M is 50 μg/mL) were evaluated (Fig. 4B, D). It was observed that compared with the control group, Cur, mPDA@M and mPDA/Cur@M/CM groups significantly induced CAFs and 4T1 cells death. Among all the groups, mPDA/Cur@M/CM + laser irradiation group displayed the lowest cell viability. These results suggested mild temperature PTT combined with curcumin and Mn^2+^ could effectively kill the tumor cells and display synergetic effects.

Next, the calcein acetoxymethyl ester (calcein-AM)/propidium iodide (PI) staining was used to determine the survival status of CAFs and 4T1 cells. Among all the groups without laser irradiation, both CAFs and 4T1 cells treated with mPDA/Cur@M/CM displayed significantly higher cell death than that of Cur and mPDA/Cur@M group (Fig. 4E, F). Interestingly, the red fluorescence intensity of CAFs and 4T1 cells in the mPDA/Cur@M/CM + laser irradiation group was found to be remarkably higher than that of mPDA/Cur@M/CM group, which indicated that the introduction of mild phototherapy could effectively kill tumor cells. Moreover, the death rate of 4T1 cells treated with mPDA/Cur@M/CM + laser irradiation was markedly higher than that of CAFs cells. Therefore, the above results confirmed that mPDA/Cur@M/CM + laser irradiation mediated mild phototherapy and cytotoxic free radicals can achieve enhanced cell-killing effects, especially with 4T1 cells.

To further investigate the mechanisms of mild phototherapy mediated anti-tumor effect, the expression levels of HSP90 in CAFs and 4T1 cells after different treatments were analyzed (Fig. 4G-F). It was observed that in comparison with the control group, the expression level of HSP90 in both CAFs and 4T1 cells was increased after treatment with mPDA@M + laser. On the contrary, the expression levels of HSP90 in Cur and mPDA/Cur@M/CM group with and without the laser irradiation were significantly decreased in both kinds of cells. These results substantiated that the introduction of curcumin and mPDA/Cur@M/CM could significantly weaken the resistance of tumor cells to thermal injury *via* reducing the expression of HSP90, and lead to substantial satisfactory anti-tumor effects under low temperature conditions, which was consistent with the previous studies.

### 3.5 *In vivo* targeting and PTT ability of mPDA/Cur@M/CM

**Fig. 5:**
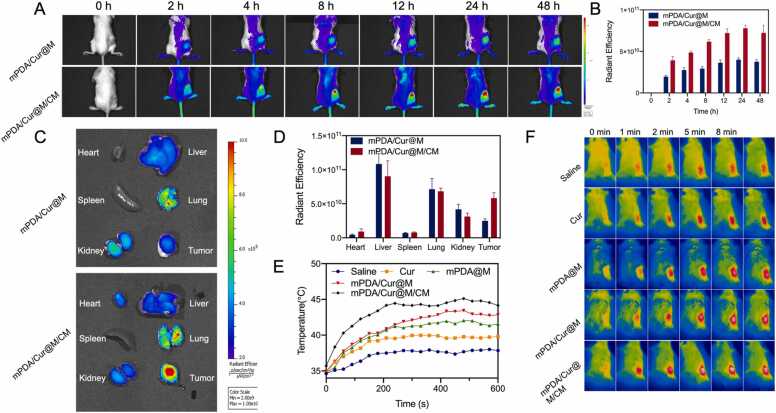
*In vivo* fluorescence images of mPDA/Cur@M and mPDA/Cur@M/CM (containing 20 μg/mL IR808) at different time point (A) and corresponding quantitative analysis of fluorescence intensity (B). *Ex vivo* fluorescence images of major organs and tumor after 48 h post-injection (C) and corresponding quantitative analysis of fluorescence intensity (D). Temperature profiles (E) and corresponding infrared thermal images (F) of tumors during 10 min of laser irradiation (808 nm, 0.4 W/cm^2^).

In order to assess the *in vivo* targeting ability of mPDA/Cur@M/CM, IR808 was used as a model drug to visualize tumor accumulation. The fluorescence signal of mPDA/IR808@M/CM from the tumor tissue emerged after 2 h administration, which continued to increase gradually, finally reached a peak at 24 h, and existed even at 48 h after administration (Fig. 5A, B). However, the fluorescent signal of tumor in mPDA/IR808@M group was significantly weaker than that of mPDA/IR808@M/CM group, thereby indicating that mPDA/IR808 @M could not specifically accumulate in the tumor tissues. At the end of the experiment, the mice were anesthetized and the major organs as well as tumor tissues were removed for fluorescent imaging (Fig. 5C, D). The fluorescent signal of *ex vivo* tumor in mPDA/Cur@M/CM group was found to be 2.34 times higher than that of mPDA/Cur@M group. These results confirmed that mPDA/Cur@M/CM with hybrid cell membrane modification was endowed with good targeting ability and could facilitate selective accumulation in the specific tumor tissues. Considering the prominent anti-tumor efficacy of mPDA/Cur@M/CM plus laser irradiation *in vitro*, the *in vivo* tumor inhibition ability was further explored on CAFs and 4T1 tumor-bearing mice. To evaluate the potential PTT effect of mPDA/Cur@M/CM, laser irradiation (808 nm, 0.4 W/cm^2^, 10 min) was performed 12 h after administration and *in vivo* temperature curves as well as the near infrared thermal images were recorded (Fig. 5E, F). The temperature of the tumor tissue in mPDA/Cur@M/CM group rapidly increased from 35.7 °C to 42.3 °C after 2 min of laser irradiation. This temperature was able to meet the subsequent requirements of mild PTT of tumor.

### 3.6 Anti-tumor efficiency of mPDA/Cur@M/CM

**Fig. 6:**
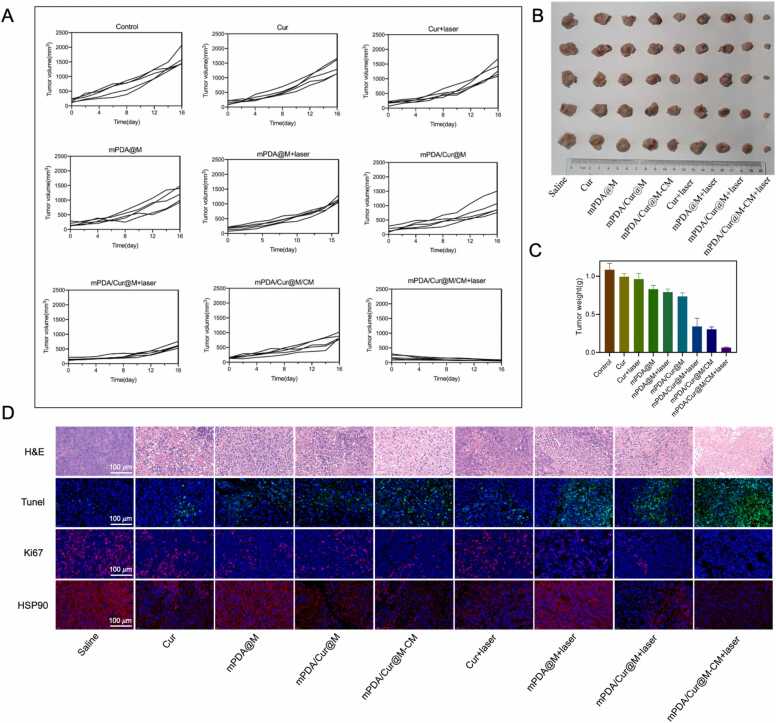
Anti-tumor ability of mPDA/Cur@M/CM. (A) The volume of tumors with different treatments (n = 6 per treatment, curcumin = 20 mg/Kg, mPDA = 25 mg/kg). (B) The body weight of multicell tumor-bearing female BALB/c mice with different treatments. (C) The images of *ex vivo* tumors. (D) Histological analysis of tumors by H&E staining, and Tunel, Ki67 and HSP90 detection by immunofluorescence staining after different treatments, scale bar = 100 µm.

*In vivo* anti-tumor efficacy of mPDA/Cur@M/CM was then examined in CAFs and 4T1 multi-cells tumor-bearing mice. The tumors in the saline group grew rapidly during the treatment, whereas the growth of tumor in other groups showed varying degrees of inhibition (Fig. 6A). When treated with curcumin with or without laser irradiation, tumor growth was just slightly inhibited. However, in comparison with the control group, the tumor growth of the mice treated with mPDA@M were significantly inhibited. This could be attributed to the substantial reduction of manganese dioxide shell by GSH in tumor microenvironment to Mn^2+^, which further mediates Fenton like reaction, thus inhibiting the tumor growth through chemodynamic therapy. Additionally, the growth of tumor in mPDA/Cur@M group showed a higher degree of inhibition than that of mPDA@M, thereby indicating curcumin loaded in the mPDA@M can successfully accumulate at the tumor site and play an anti-tumor role through exhibiting cytotoxic effects. Notably, treatment with mPDA/Cur@M/CM + laser irradiation could lead to a greater anti-tumor effect than that of mPDA/Cur@M + laser irradiation and mPDA/Cur@M/CM, suggesting that both the mild photothermal effect and the introduction of cell membrane modification were essential for enhanced anti-tumor efficacy. The images of resected tumors have been shown showed in Fig. 6B and the final tumors weight at the end of treatment were consistent with the tumors growth (Fig. 6C). These findings illustrated that the combination of mPDA mediated mild photothermal effect, curcumin mediated cytotoxicity and Mn^2+^ mediated Fenton-like reaction can exhibit synergistic effect, which could be useful to obtain better anti-tumor effect.

To further evaluate the anti-tumor efficacy and explore the mechanism of mPDA/Cur@M/CM from the tissue level, the hematoxylin and eosin (H&E), TUNEL and ki67 staining of tumor tissues were performed and the expression of HSP90 protein was detected. For H&E staining, the structure of tumor tissue in the mPDA/Cur@M/CM + laser irradiation group was found to be substantially reduced or even disappeared, and a severe necrosis was observed (Fig. 6D). Then, terminal deoxynucleotidyl transferase-mediated dUTP-biotin nick end labeling (TUNEL) staining was utilized to directly evaluate the possible anti-tumor effect of different treatment. In addition, compared with other groups, greater extent of green fluorescence signals were observed in the mPDA/Cur@M/CM + laser irradiation group, indicating that higher extent of necrosis and apoptosis, which could be attributed to the thermal damage of mild PTT and the cytotoxicity of curcumin and Mn^2+^ (Fig. 6D). Similarly, ki67 was used to determine the proliferative ability of the tumor cells. The mPDA/Cur@M/CM + laser irradiation group exhibited weaker fluorescence signals than other groups, indicating less extent of proliferation, which was consistent with the results of TUNEL staining. Next, the expression level of HSP90 protein was detected to analyze the heat resistance of the tumor cells. The red fluorescence signals was significantly decreased in the mPDA/Cur@M/CM + laser irradiation group, thereby revealing that the down regulated expression of HSP90 protein induced poor heat tolerance which was would conducive for achieving better anti-tumor efficacy. In conclusion, mild PTT combined with chemotherapy could effectively promote tumor cell apoptosis and inhibit tumor proliferation.

### 3.7 *In vivo* biosafety evaluation of mPDA/Cur@M/CM

The body weight of the mice were monitored to determine biosafety of the different treatment regimens. The result showed that the body weight remained steady and there was no significant difference among the nine distinct groups, demonstrating nanoparticles synthesized in this study caused minimal systemic toxicity (Fig. S6). To verify the *in vivo* biosafety of Cur, mPDA@M, mPDA/Cur@M and mPDA/Cur@M/CM with or without laser irradiation, the routine blood, liver function and kidney function were examined on day 16 after the treatment. The values of WBC, RBC, HGB, PLT of all the groups showed no significant differences, indicating that there was no obvious abnormality in the hematopoietic function of bone marrow (Fig. S7). Additionally, the values of AST, ALT, BUN and CRE of all groups exhibited no remarkable abnormalities, thus demonstrating all treatments in this work could not cause damage to liver and kidney function (Fig. S8). To further substantiate the biosafety of Cur, mPDA@M, mPDA/Cur@M and mPDA/Cur@M/CM nanoparticles in the presence of or absence of the laser irradiation, H&E staining of the main organs (heart, liver, spleen, lung and kidney) were performed. According to the Fig. S9, no visible damage was found in these organs, which further indicated that the multifunctional nanoparticles constructed in this work exhibited good biosafety.

## 4 Conclusion

To sum up, we have successfully synthetized a novel multifunctional nano system (mPDA/Cur@M/CM) for drug sequential delivery and synthetic treatment. The results showed that multicell membrane-modified nanoparticles were endowed with homology target ability, resulting in precise accumulation in tumor sites. Moreover, thermal damage induced by mild PTT combined with cytotoxicity induced by curcumin and Mn^2+^ could serve as a safe and effective strategy for TNBC treatment. mPDA can effectively transform the power of laser to thermal energy and further perform mild PTT on the tumor cells. In addition, curcumin can effectively inhibit HSP90 expression, remarkably reduce tumor cell heat tolerance, and facilitate effective PTT even at the mild temperature. At the same time, curcumin mediated cytotoxicity and Mn^2+^ mediated Fenton like reaction could induce apoptosis of tumor cells. Overall, mPDA/Cur@M/CM can precisely deliver drugs into the tumor tissues through multicell membrane mediated orderly targeting, and further achieve enhanced anti-tumor efficiency through combining mild photothermal with chemotherapy. The findings of this study shed light on the development of safer and efficient strategies for TNBC treatment.

## CRediT authorship contribution statement

L.W., Q.L., A.W. and T.C. conceived and initiated the research. L.W. and M.W. synthesized nanoparticles. L.W., M.W., Y.P., X.D., C.H., J.L. X.M. H.L. and H.L. were responsible for all experiments. M.W. and T.C. wrote the manuscript. All authors analyzed the data and have given approval to the final version of the manuscript.

## Declaration of Competing Interest

The authors declare that they have no known competing financial interests or personal relationships that could have appeared to influence the work reported in this paper.
