# Supplementary material for: Sequential targeting biomimetic nano platform for enhanced mild photothermal therapy and chemotherapy of tumor
Source: Comput Struct Biotechnol J. 2023 Apr 26;21:2780–91. doi: 10.1016/j.csbj.2023.04.024 (PMC10172638; doi:10.1016/j.csbj.2023.04.024)
Supplement: Supplementary file 1 — Supplementary material [file mmc1.docx]

**Supplementary Information**

**Sequential targeting biomimetic nano platform for enhanced mild photothermal therapy and chemotherapy of tumor**

*Lianfu Wang^1,2†^, Manxiang Wu^1,2†^,^，^Yuning Pan^3†^, Dong Xie^1^, Chengyuan Hong^2,4^, Jianbin Li^1^, Xuehua Ma^2^,Huachun Xu^1^, Huayu Li^1^, Tianxiang Chen^2*^, Aiguo Wu^2*^, Qiang Li^1*^*

1. Department of Radiology, The Affiliated People’s Hospital, Ningbo University, Ningbo, 315040, China.

2. Cixi Institute of Biomedical Engineering, International Cooperation Base of Biomedical Materials Technology and Application, Chinese Academy of Science (CAS) Key Laboratory of Magnetic Materials and Devices, Zhejiang Engineering Research Center for Biomedical Materials, Ningbo Institute of Materials Technology and Engineering, CAS, 1219 Zhongguan West Road, Ningbo 315201, P. R. China. Advanced Energy Science and Technology Guangdong Laboratory, Huizhou 516000, P.R. China

3. Department of Radiology, Ningbo First Hospital, Ningbo, 315010, China.

4. Department of Mechanical, Materials and Manufacturing Engineering, University of Nottingham Ningbo China, Ningbo, 315100, P.R. China

.

* Corresponding Authors

E-mail addresses: [rmliqiang@nbu.edu.cn](mailto:rmliqiang@nbu.edu.cn) (Qiang Li), aiguo@nimte.ac.cn ( Aiguo Wu) and chentx@nimte.ac.cn (Tianxiang Chen)

† These authors contributed equally.

*
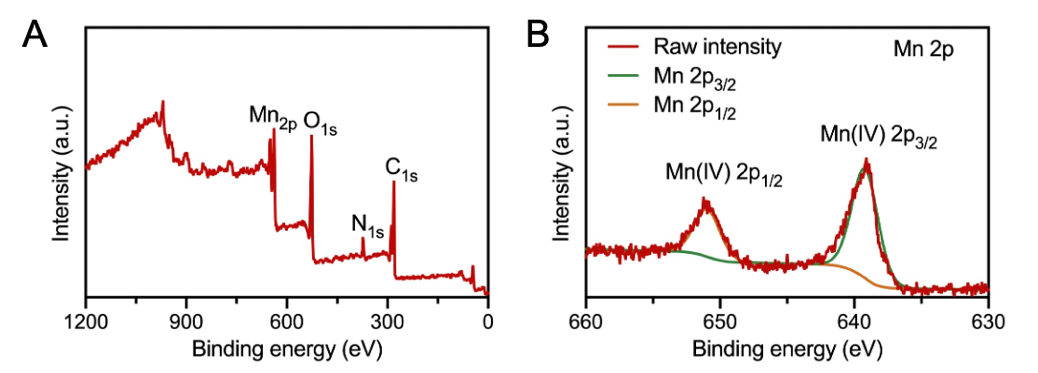
*

Figure S1. The XPS curve (A) and Mn 2p XPS spectrum (B) of mPDA/Cur@M.


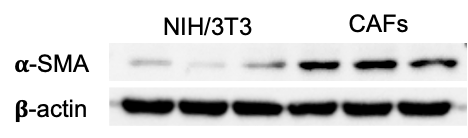


Figure S2. The 𝛼-SMA expression level of NIH/3T3 cells before and after TGF-𝛼 treatment.


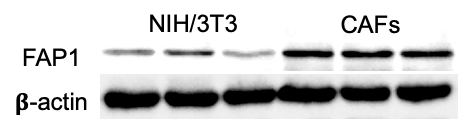


Figure S3. The FAP1 expression level of NIH/3T3 cells before and after TGF-𝛼 treatment.


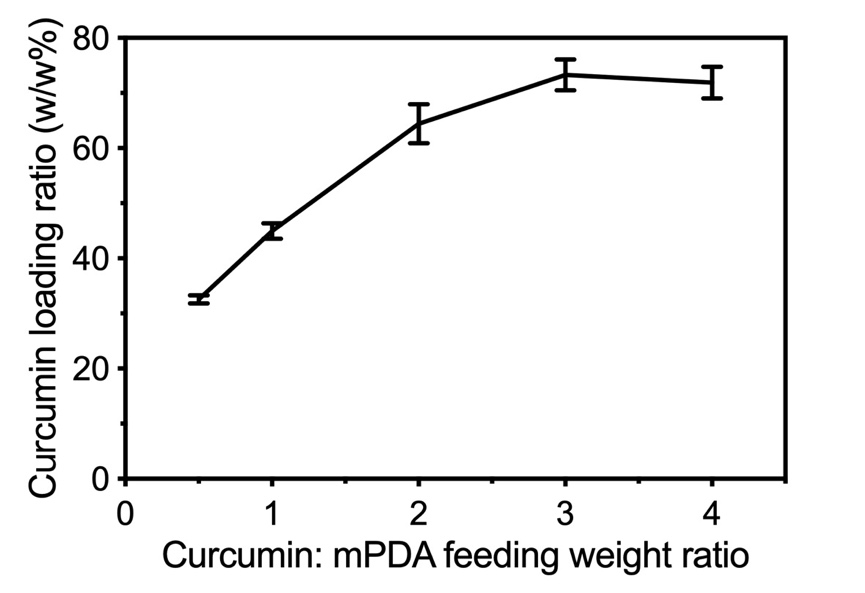


Figure S4. Curcumin loading weight ratio of mPDA/Cur@M/CM at different drug feeding weight ratios.


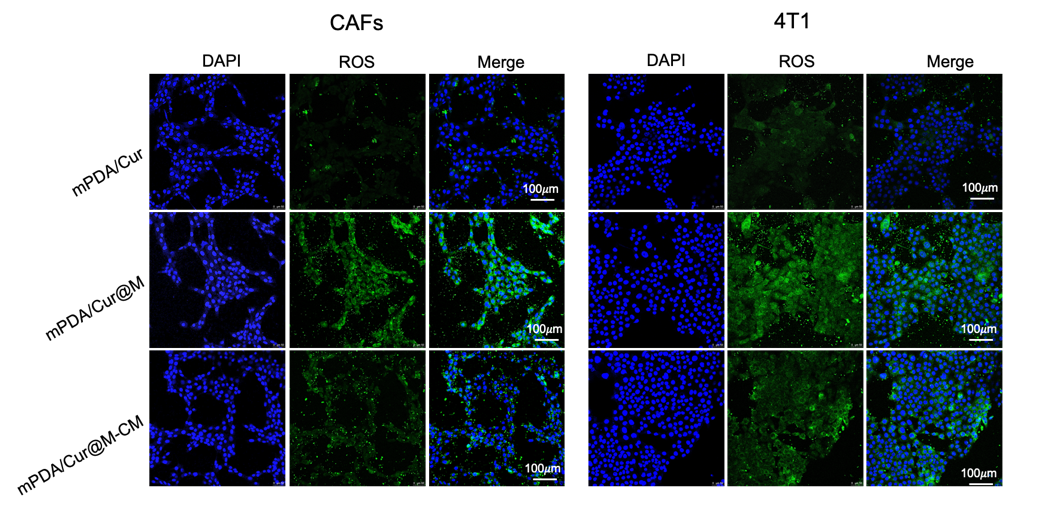


Figure S5. The fluorescence images of ROS (green) and nucleus (blue) in CAFs and 4T1 cells with different treatments.


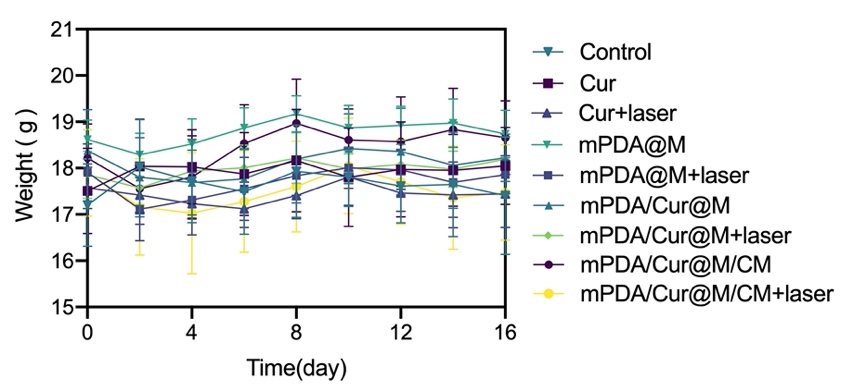


Figure S6. The body weight of mice during the whole treatment process.


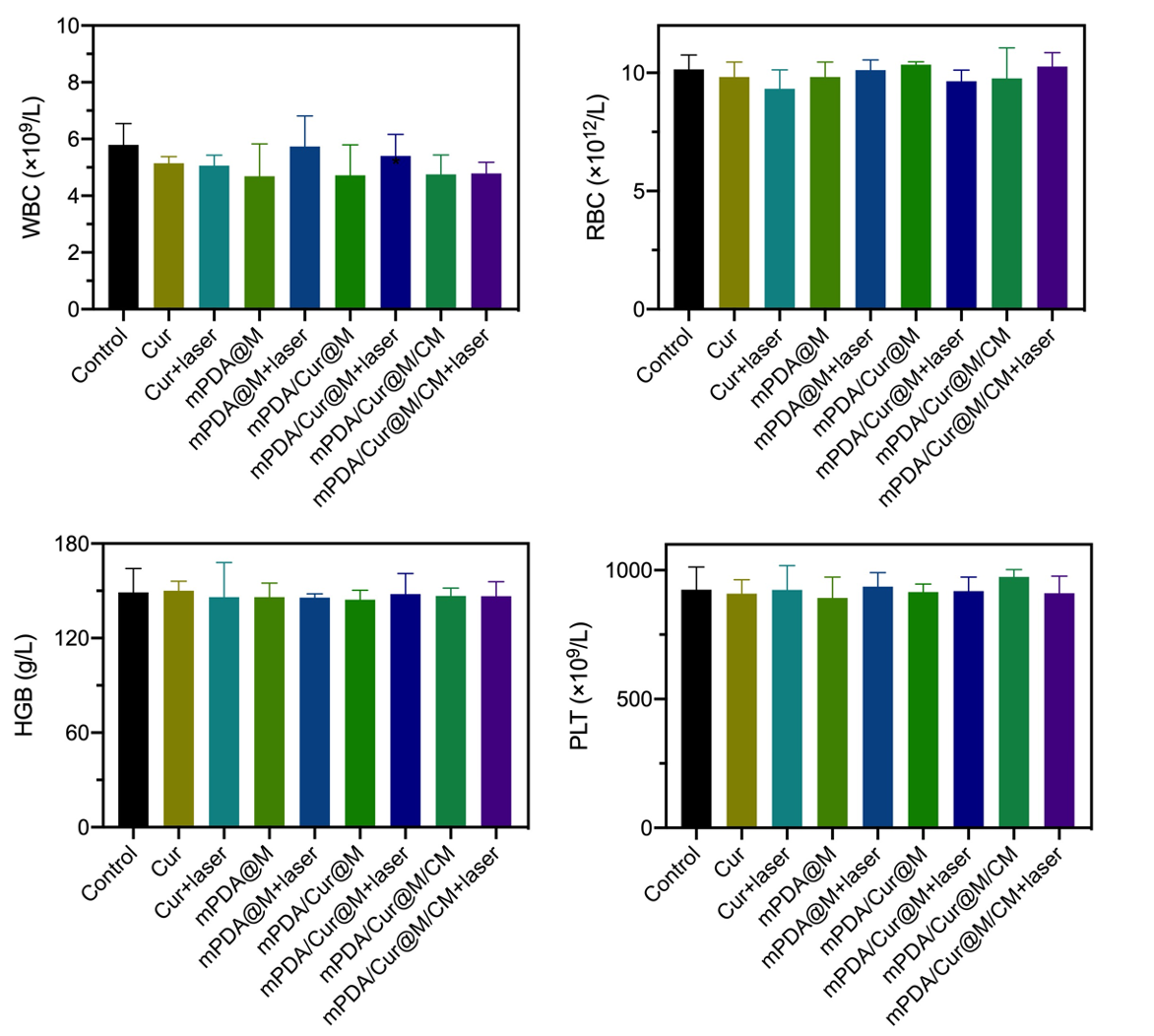


Figure S7. Blood routine assay of mice after different treatments. The WBC, RBC, HGB and PLT level of mice treated with Saline, Cur, Cur + laser, mPDA@M, mPDA@M + laser, mPDA/Cur@M，mPDA/Cur@M + laser，mPDA/Cur@M/CM and mPDA/Cur@M/CM + laser at the end of treatment. n=4 (808 nm, 0.4 W/cm^2^, 10 min).


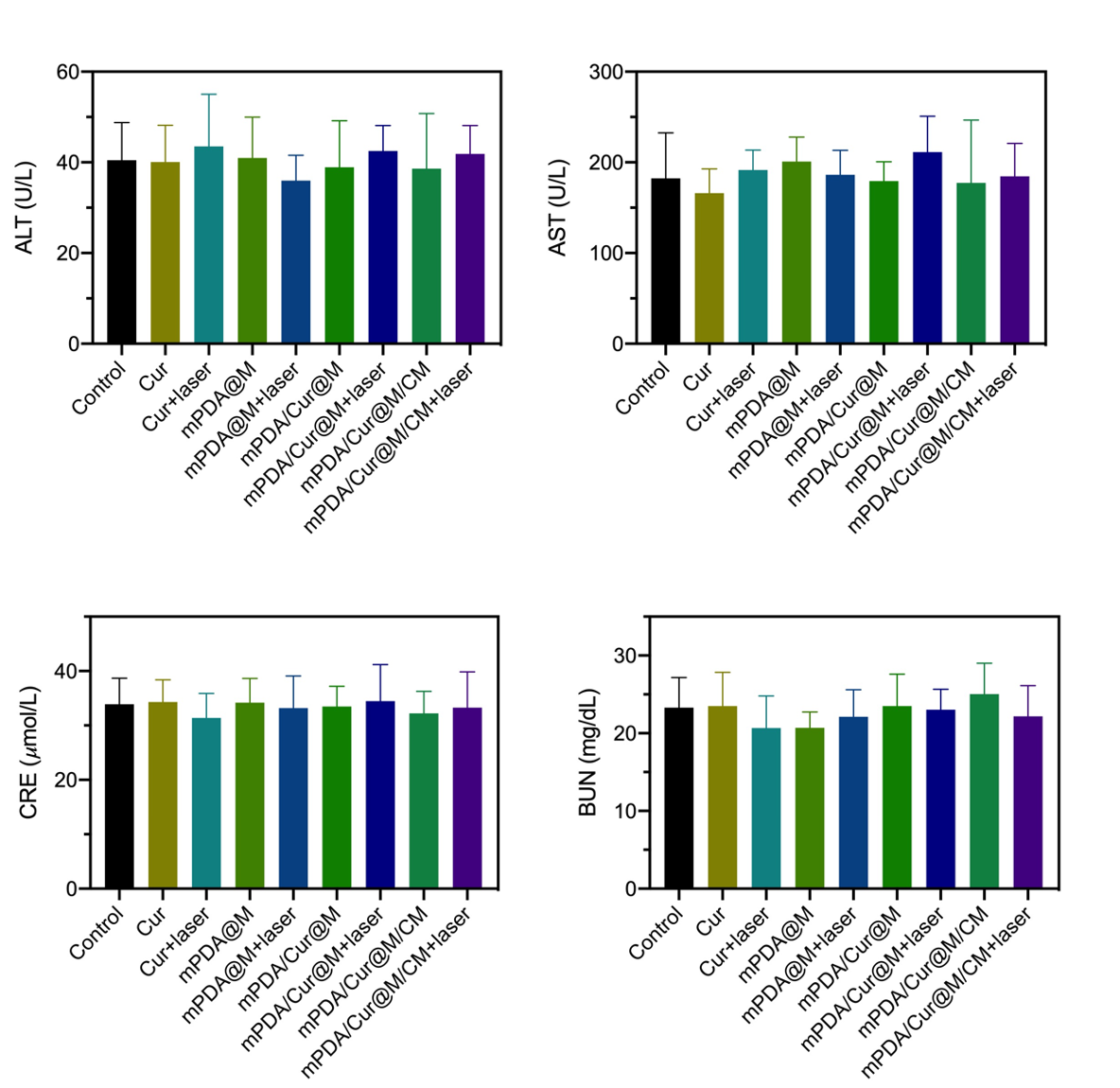


Figure S8. Liver and kidney function assay of mice after different treatments. The ALT, AST, CRE and BUN level of mice treated with Saline, Cur, Cur + laser, mPDA@M, mPDA@M + laser, mPDA/Cur@M，mPDA/Cur@M + laser，mPDA/Cur@M/CM and mPDA/Cur@M/CM + laser at the end of treatment. n=4 (808 nm, 0.4 W/cm^2^, 10 min).


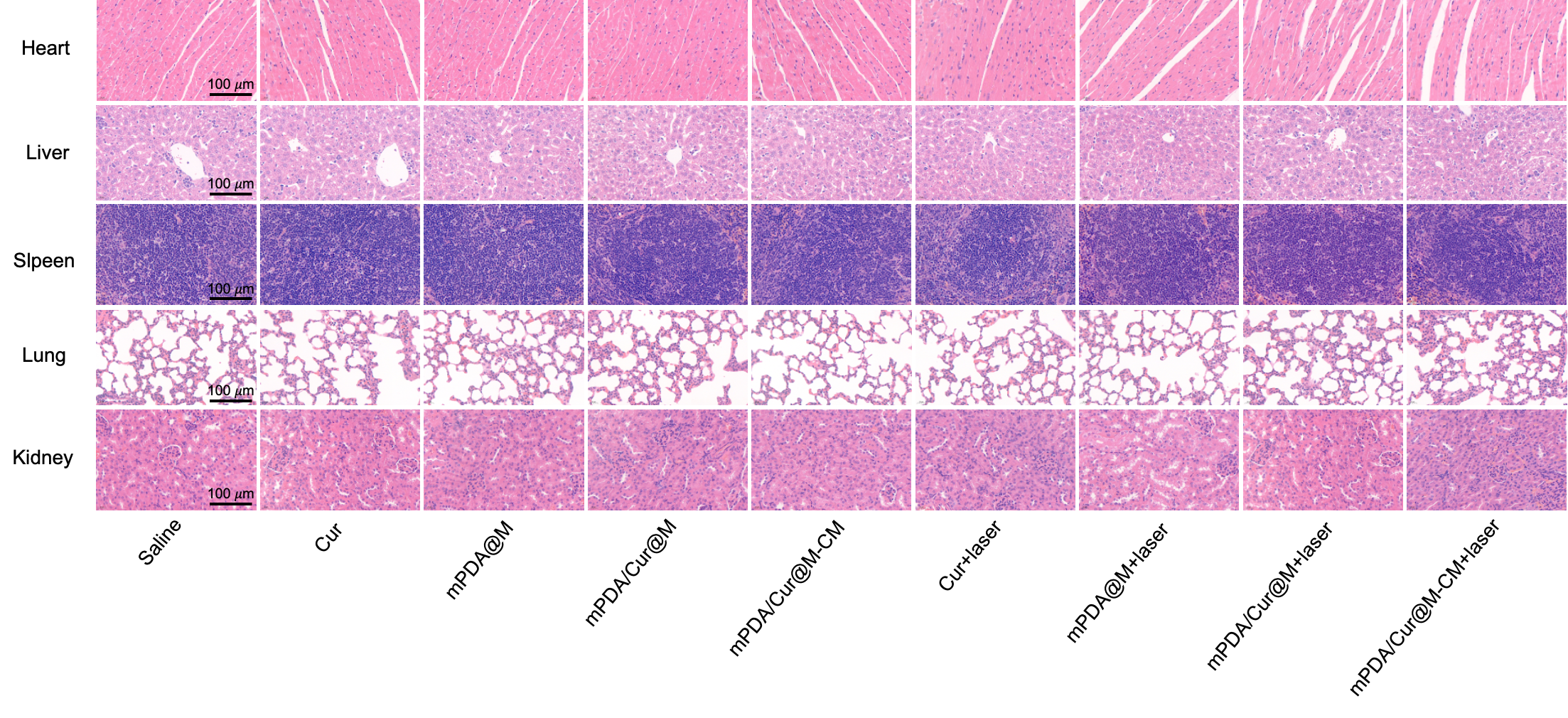


Figure S9. H&E staining of the major organs from 4T1-bearing mice after different treatments. The bar indicates 100 μm.
